# Cell-autonomous and differential endocannabinoid signaling impacts the development of presynaptic retinal ganglion cell axon connectivity *in vivo*

**DOI:** 10.3389/fnsyn.2023.1176864

**Published:** 2023-05-12

**Authors:** Rodrigo Del Rio, Rosa G. Serrano, Eric Gomez, Joshua C. Martinez, Marina A. Edward, Rommel A. Santos, Kenneth S. Diaz, Susana Cohen-Cory

**Affiliations:** Department of Neurobiology and Behavior, University of California, Irvine, Irvine, CA, United States

**Keywords:** *Xenopus laevis*, live imaging, optic tectum, knockdown, anandamide (AEA), 2-Arachidonoylglycerol (2-AG)

## Abstract

Cannabis exposure during gestation evokes significant molecular modifications to neurodevelopmental programs leading to neurophysiological and behavioral abnormalities in humans. The main neuronal receptor for Δ^9^-tetrahydrocannabinol (THC) is the type-1 cannabinoid receptor CB_1_R, one of the most abundant G-protein-coupled receptors in the nervous system. While THC is the major psychoactive phytocannabinoid, endocannabinoids (eCBs) are the endogenous ligands of CB_1_R and are known to act as retrograde messengers to modulate synaptic plasticity at different time scales in the adult brain. Accumulating evidence indicates that eCB signaling through activation of CB_1_R plays a central role in neural development. During development, most CB_1_R localized to axons of projection neurons, and in mice eCB signaling impacts axon fasciculation. Understanding of eCB-mediated structural plasticity during development, however, requires the identification of the precise spatial and temporal dynamics of CB_1_R-mediated modifications at the level of individual neurons in the intact brain. Here, the cell-autonomous role of CB_1_R and the effects of CB_1_R-mediated eCB signaling were investigated using targeted single-cell knockdown and pharmacologic treatments in *Xenopus*. We imaged axonal arbors of retinal ganglion cells (RGCs) in real time following downregulation of CB_1_R *via* morpholino (MO) knockdown. We also analyzed RGC axons with altered eCB signaling following treatment with URB597, a selective inhibitor of the enzyme that degrades Anandamide (AEA), or JZL184, an inhibitor of the enzyme that blocks 2-Arachidonoylglycerol (2-AG) hydrolysis, at two distinct stages of retinotectal development. Our results demonstrate that CB_1_R knockdown impacts RGC axon branching at their target and that differential 2-AG and AEA-mediated eCB signaling contributes to presynaptic structural connectivity at the time that axons terminate and when retinotectal synaptic connections are made. Altering CB_1_R levels through CB_1_R MO knockdown similarly impacted dendritic morphology of tectal neurons, thus supporting both pre- and postsynaptic cell-autonomous roles for CB_1_R-mediated eCB signaling.

## Introduction

About 2.5–5.0 percent of pregnant women in the US report using cannabis during gestation, yet little is known about the effects of cannabinoid exposure during pregnancy (Henschke, 2019). Various large-scale longitudinal linkage studies have demonstrated potential consequences that include low birth weight, impulsivity and hyperactivity in children, and higher rates of anxiety, depression and in drug abuse later in life (Calvigioni et al., 2014). Similarly, maternal cannabinoid exposure during lactation may impact brain development postnatally due to its high concentration in breast milk, eight times that of a mother’s plasma levels (Henschke, 2019). It is therefore important to have a clear understanding of the roles of endocannabinoids (eCBs) and their receptors during early neuronal differentiation to better understand the biological mechanisms involved in cannabis use and the functional consequences of cannabinoid exposure on the developing brain. A large number of studies have shown that eCBs have critical functions in fetal and postnatal brain development, neuronal connectivity, and glial cell differentiation (Berghuis et al., 2007; Harkany et al., 2007, 2008a,b; Martinez et al., 2020; Marinelli et al., 2023). eCBs are important neuromodulators of multiple central neurotransmitter systems that are essential for fetal brain development (Gaffuri et al., 2012). eCBs released from post-synaptic neurons serve as retrograde signals that suppress neurotransmitter release at cortical synapses (Freund et al., 2003), but also can act in a non-retrograde and/or autocrine manner to modulate synaptic function and can interact with other neuromodulatory systems (Castillo et al., 2012). Dissociated cell cultures, mouse, and zebrafish models with targeted deletion of the cannabinoid receptor type-1 (CB_1_R) have shown that neurite extension and axon fasciculation are affected by altered cannabinoid signaling (Mulder et al., 2008; Watson et al., 2008; Wu et al., 2010). In the visual system, pharmacological activation or blockade of CB_1_R activity can modulate growth cone morphology of cortical and retinal neurons in culture and affects the topographic organization of retinal projections in the brain of knockout mice (Argaw et al., 2011). Because eCBs dynamically regulate brain development, the timing and duration of cannabinoid exposure may have differential effects on developing neurons, and long-term consequences may vary depending on the cellular context. Studies are therefore needed to characterize roles of eCB signaling at multiple stages of brain and neural development in intact organisms especially given that exogenously administered cannabinoids possess medicinal properties and their recreational and therapeutic use in the management of nausea and vomiting during pregnancy is on the rise (Henschke, 2019). Here, we characterized cellular mechanisms by which eCB signaling modulates neural circuit development *in vivo* by differentiating cell-autonomous effects versus global effects of pharmacologic treatments. Moreover, our *in vivo* single-cell analysis allowed us to distinguish between the influence of cannabinoids on developing synaptic circuits at distinct times of development.

Endocannabinoids (eCBs) are lipophilic molecules, which are thought to be synthesized on demand from plasma membrane components through multiple biosynthetic pathways (Freitas et al., 2018). Dietary polyunsaturated fatty acids (PUFAs) serve as precursors for eCBs in the brain. Arachidonic acid (AA), the principal n-6 PUFA in the brain, is produced from linoleic acid (LA), and serves as the precursor to anandamide (AEA), an important eCB that has been implicated in several aspects of neural development (Harkany et al., 2007; Maccarrone et al., 2014; Anderson et al., 2015). Similar to AEA, docosahexaenoic acid (DHA, 22:6n-3), a n-3 polyunsaturated fatty acid (n-3 PUFA) and an essential component of the central nervous system (CNS), is also synthesized from dietary precursors such as α-linolenic acid (ALA) (Freitas et al., 2018). Previous work from our laboratory assessed how depriving such dietary PUFAs from adult maternal *Xenopus* frogs affects retinotectal development. Using *Xenopus* as a model, we demonstrated that maternal n-3 PUFA deficiency significantly alters the morphology and early connectivity of developing central neurons in the progeny, coincident with reduced embryonic brain DHA levels and a decrease in BDNF-mediated neurotrophic support (Igarashi et al., 2015). Maternal dietary DHA supplementation was able to rapidly reverse the n-3 PUFA mediated developmental deficits (Igarashi et al., 2015). AEA, similarly to DHA derivatives in the brain, is synthesized by the N-acyl phosphatidylethanolamine-specific phospholipase D (NAPE-PLD) and hydrolyzed by fatty acid amide hydrolase (FAAH). Another major eCB that can also bind CB_1_R with high affinity is 2-Arachidonoylglycerol (2-AG), synthetized by hydrolysis from diacylglycerols (DAGs) by two lipases, DAGLα and DAGLβ, and hydrolyzed by monoacylglycerol lipase (MAGL). Here, we used the relative simplicity of the *Xenopus* visual system to examine basic underlying cellular mechanisms by which n-6 PUFA-derived eCBs modulate presynaptic connectivity. We explored how altered cannabinoid signaling can adversely affect presynaptic retinal ganglion cell (RGC) axons at distinct epochs of development, specifically during axon targeting and during the development of structural and functional brain connectivity. By examining dynamic cellular changes in presynaptic connectivity in postmitotic neurons we were able to differentiate effects of endocannabinoids in the intact, developing brain, and to correlate structural with functional changes.

## Materials and methods

### Animals

*Xenopus laevis* tadpoles were obtained by either *in vitro* fertilization of oocytes or by natural mating of adult females primed with human chorionic gonadotropin. Female and male pairs were left to mate overnight and embryos were collected following 12 h post-injection. Tadpoles were raised in rearing solution [60 mM NaCl, 0.67 mM KCl, 0.34 mM Ca(NO_3_)_2_, 0.83 mM MgSO_4_, 10 mM HEPES, pH 7.4, and 40 mg/l gentamicin] plus 0.001% phenylthiocarbamide to halt melanocyte pigmentation, with a 12-h light and dark cycle. Tadpoles from stage 38/39 (2.5 to 3 days post-fertilization at 20°C) to stage 45 (5 days post-fertilization at 20°C) were anesthetized during experimental manipulations with 0.05% tricaine methanesulfonate (Finquel; Argent Laboratories, Redmond, WA, USA). Staging of embryos was performed according to Nieuwkoop and Faber (1956). The sex of tadpoles used for experimentation and analyses was random and unknown, as gonadal differentiation in *Xenopus laevis* begins well after stage 49 (Piprek et al., 2017), 9 to 13 days postfertilization. Animal procedures were approved by the Institutional Animal Care and Use Committee of the University of California, Irvine (Animal Welfare Assurance Number A3416–01).

### Immunohistochemistry

Stage 42 to stage 47 tadpoles were euthanized with tricaine methanesulfonate and fixed in 4% paraformaldehyde in 0.1 M phosphate buffer, pH 7.5, for 2 h. For coronal sections, tadpoles were cryoprotected in 30% sucrose overnight and embedded in OCT compound (Sakura Finetek, Torrance, CA, USA), and 25-μm cryostat sections were obtained. Coronal sections at the level of the optic tectum were incubated with a rabbit polyclonal antibody against a synthetic peptide from the N-terminal extracellular region of human CB_1_ receptor (1:200 dilution; Cayman Chemicals) or a rabbit polyclonal antibody against the cannabinoid receptor CB_1_(1–77) (1:250 dilution, Cat# 209550, Calbiochem). CB_1_R primary antibodies were visualized using goat anti-rabbit Alexa 488 secondary antibodies (1:500 dilution; Invitrogen, Eugene, OR, USA). The specificity of CB_1_R antibodies (1:500 dilution) to recognize endogenous *Xenopus* CB_1_R was previously tested and confirmed by Western blot analysis: a band of∼60 kDa was detected by anti- CB_1_R antibodies in stage 45 *Xenopus* brain lysates similar to the chick brain (da Silva Sampaio et al., 2018).

### Transfection of morpholinos or plasmids

Downregulation of CB_1_R expression was performed using lissamine-tagged morpholino anti-sense oligonucleotides (300 nmol, Genetools, Philomath, OR, USA) to block protein translation in *Xenopus* tadpoles similar to published studies (Santos et al., 2018), a treatment that results in 40–60% reduction in protein levels. A morpholino (MO) against *Xenopus laevis* cannabinoid receptor 1 *Cnr1* mRNA was designed with the sequence 5′-GGCCATCCAGAATTGACTTCATTAC-3′ as described in Zheng et al. (2015). A standard lissamine-tagged control morpholino oligonucleotide with the following sequence 5′-CCTCTTACCTCAgTTACAATTTATA-3′ with no known targeting effects was used for control comparisons. Targeted downregulation of CB_1_R expression in developing RGCs or in tectal neurons was achieved using single-cell electroporation in developing *Xenopus* tadpoles (Liu and Haas, 2011). Prior to electroporation, tadpoles were anesthetized with 0.05% tricaine methanesulfonate. A CUY-21 edit stimulator was used to electroporate and transfect individual RGCs or tectal neurons of stage 42–43 tadpoles. RGCs or tectal neurons were co-electroporated with lissamine-tagged CB_1_R MO (150 nmol pipette concentration) and a cell-filling dye Alexa Fluor 488 dextran, 3,000 MW (2 mg/111 μl pipette concentration, Invitrogen, Eugene, OR, USA), with one electroporation performed per tadpole. Reagents were loaded onto an aluminosilicate pipette (AF100-64-10, 1.00 mm, 0.64 mm, 10 cm) with a pulled tapered-tip with an opening of about 0.5 μm. Neurons transfected with a standard lissamine-tagged control MO (100 nmol pipette concentration) and Alexa Fluor 488 dextran were used as a control comparison with CB_1_R MO transfected neurons. Tadpoles were then raised at 22°C until stage 45 (1 day later). Co-transfections of lissamine-tagged morpholinos and Alexa 488 dextran were confirmed *via* fluorescence microscopy. In some tadpoles, leaky single-cell electroporations resulted in more than one transfected neuron. Only tadpoles with individual RGC axons or tectal neurons double-labeled with lissamine and Alexa 488 dextran in the optic tectum were selected for two-photon confocal imaging and analysis (Santos et al., 2018).

### Transfection of RGCs with plasmids by electroporation to visualize axon arbors and presynaptic sites

A 2–5 μL mix of Green fluorescent protein (GFP)-synaptobrevin and tdTomato plasmids at equimolar amounts (1 μg/μL) were loaded into an aluminosilicate glass capillary needle and mounted onto a three-axis manual micromanipulator. Tadpoles at stage 28–32 were anesthetized with 0.05% tricaine methanesulfonate and placed in an anesthetic-saturated Sylgard cushion with a custom-made trench. Tadpoles were mounted on their side, with the right eye up, using a standard size harp slice grid. A second micromanipulator holding a pair of cathode and anode copper electrodes were placed 0.1 mm apart to span the diameter of the eye. About 1–2 nL of DNA mix was pressure injected into the anterior chamber near the lens at 20 psi and 15 ms duration using a Picospritzer III pipet holder. A Grass SD9 stimulator was used to simultaneously deliver single currents of 40 V, 200 Hz, 2 ms delay, and 2 ms duration. Tadpoles recovered in fresh rearing solution immediately after electroporation. Tadpoles at stages 39–45 were screened and those with single RGCs expressing tdTomato and punctate GFP-syb in their axon terminals were selected and used for experimentation and imaging.

### Pharmacologic manipulations of endocannabinoid levels

We used two experimental paradigms to compare the potential effects of increased AEA signaling with those of 2-AG during RGC axon targeting, branching and presynaptic differentiation in intact animals. Swimming *Xenopus laevis* tadpoles were treated with URB597 an inhibitor of FAAH, or JZL184 a potent inhibitor of MAGL-2, at two distinct stages of visual system development; at stage 38–39 when RGC axons target the optic tectum and at stage 45, when they begin to actively branch. Tadpoles starting at stage 38–39 were reared in multi-well plates in the presence of either URB597 (2.5 μM in 0.1% Dimethyl sulfoxide (DMSO) in rearing solution; Cayman Chemicals, Ann Arbor, MI, USA), JZL184 (2.5 μM in 0.1% DMSO in rearing solution; Cayman Chemicals), or vehicle solution (0.1% in DMSO) for a total of 3 days (until stage 45) in the presence of the drug, with replenishment of fresh pharmacologic agent and rearing solution every 24 h. The concentration of the pharmacologic agents used is within the lowest concentration range shown to be effective and elicit differential effects on sensorimotor function in *Xenopus* tadpoles and zebrafish larvae (Miraucourt et al., 2016; Khara et al., 2022). Tadpoles were then imaged *in vivo* every 24 h for 3 days beginning at stage 45 and reared between observation intervals in the absence of the drug treatment. For pharmacologic treatment of tadpoles beginning at stage 45, tadpoles with single RGCs expressing tdTomato and punctate GFP-synaptobrevin in their axon terminals were first imaged (0 h) and then immediately transferred to fresh rearing solution with either vehicle (0.1% DMSO), URB597 (2.5 μM), or JZL184 (2.5 μM). Tadpoles were reared for 24 h in the presence of the drug and then anesthetized to obtain a second imaging time point (24 h). After the second imaging, tadpoles were returned to fresh rearing solution with fresh drug, reared for 24 h and then imaged (48 h imaging time point). In a subset of experiments aimed to assess the acute effects of drug treatment, stage 45 tadpoles with single RGCs expressing tdTomato and punctate GFP-synaptobrevin in their axon terminals were anesthetized after the initial imaging and 1 nL of vehicle solution or 1 nL of a 50 μM solution of URB597 was injected into the ventricle and lateral side of the tectal neuropil. Tadpoles were then transferred to fresh rearing solution for recovery and were then imaged 6, 12 and 24 h after initial imaging, at stage 45.

### Fatty acid analysis of tadpole samples

A total of twenty tadpoles per experimental condition, either at stage 38–39 or at stage 45, were treated as above with either URB597 (2.5 μM in 0.1% DMSO in rearing solution; Cayman Chemicals, Ann Arbor, MI, USA), JZL184 (2.5 μM in 0.1% DMSO in rearing solution; Cayman Chemicals), or vehicle solution (0.1% DMSO) for 1 h. Tadpoles were then transferred to fresh rearing solution without the drug, anesthetized in 0.05% Finquel, quickly rinsed to remove most of the rearing solution containing the drug, and immediately frozen until further processing for fatty acid analysis. Frozen tadpole samples were homogenized, extracted and processed for liquid chromatography-mass spectrometry (LC/MS-MS) analyses as in Torrens et al. (2023). Triplicate experiments were used for the statistical analysis of data. One-way and two-way ANOVA with Tukey’s multiple comparison tests were used to statistical analysis of data. Results were considered significant in comparison to control when *p* ≤ 0.05.

### *In vivo* real-time confocal microscopy imaging

At stage 45, tadpoles were anesthetized with 0.05% tricaine methanesulfonate and mounted in a 35 × 10-mm Petri dish containing an agar cushion (2.5% w/v agar gel in 1× MR). Tadpoles were screened with 10× and 20× objective lenses using an epifluorescence microscope for the presence of fluorescently labeled RGC axons innervating the contralateral side of the optic tectum or individual tectal neurons in the optic tectum. Tadpoles were transferred to a custom-made anesthetic-saturated Sylgard chamber and positioned in place using a standard size harp slice grid. Imaging of tadpoles co-transfected with MOs and Alexa 488 dextran was performed using an inverted laser scanning LSM780 confocal microscope (Zeiss), equipped with a MaiTai Ti: Sapphire multiphoton laser system and a 63× objective. Tadpoles were imaged over the course of 3 days, at 24-h intervals. A two-photon wavelength of 760 to 780 was used to image the Alexa 488 cell-filling dye in RGC axons or in tectal neurons in the midbrain. Neurons in tadpoles co-transfected with tdTomato and GFP-synaptobrevin were imaged using a Nikon PCM2000 laser-scanning confocal microscope equipped with Argon and HeNe lasers. To assess the effects of long-term drug treatment, tadpoles were first imaged at stage 45 and every 24 h later over the course of 2 days. To assess the acute effects of treatment, tadpoles were imaged at 6, 12 and 24 h after the initial imaging at stage 45. Images were collected in a 1 μm interval throughout the extent of the arbor. Tadpoles were allowed to recover in fresh rearing solution immediately after imaging.

### Neuronal arbor analysis

In brief, three-dimensional images of fluorescently-labeled RGC axon or tectal neuron dendritic arbors were manually reconstructed blind to treatment using the Neuromantic tracing software version 1.7.5 (Myatt et al., 2012). Each axonal or dendritic arbor was reconstructed plane-by-plane from the image z-stack and was then measured and analyzed using the Neuromantic software. Branch tips were identified as the terminal ends of primary axons or dendrites. The total arbor including branch tips was thresholded, binarized, and skeletonized with the Neuromantic software so that the arbor was represented as a single pixel width. Processes of more than 5 μm in length were considered branches, while processes less than 5 μm were categorized as filopodia (Alsina et al., 2001; Marshak et al., 2007; Manitt et al., 2009). To measure the number of GFP-synaptobrevin puncta in GFP-synaptobrevin and tdTomato double-labeled axons, overlapped images were digitized, selected for color (yellow; locations of complete red and green overlay with hue and pixel intensity values between 16–67 and 150–255, respectively) and binarized as in Alsina et al. (2001) using the MetaMorph software (Molecular Devices, Inc., San Jose, CA, USA). GFP-synaptobrevin labeled puncta of 0.5–1.0 μm^2^ in size (size of smallest puncta observed) and 150–255 pixel intensity values were considered single synaptic clusters. The number of pixels representing the GFP-synaptobrevin puncta were counted with MetaMorph (Alsina et al., 2001) and normalized by the length of the axon arbor. Similar synaptic cluster values were obtained by digital or manual counting of yellow puncta. Student’s *t*-tests and one-way ANOVA with Tukey’s or Sidak’s multiple comparison tests were used for the statistical analysis of the data. Results were considered significant in comparison to control as follows: *p ≤ 0.05, ^**^p ≤ 0.005, ^***^p ≤ 0.001, unless otherwise indicated on the graph.

### Visual avoidance task

Stage 45 tadpoles were placed in a 14 cm×15 cm glass Petri dish, with darkened walls, filled with 80 ml of modified rearing solution at room temperature. The dish was placed on a monitor screen and a solid, opaque box was placed over the monitor to eliminate outside light. A camera was affixed to the opening at the top of the box for video recording. Visual stimuli were produced by a custom-written program modified and adapted from published protocols (Dong et al., 2009; Khakhalin et al., 2014). Four tadpoles were placed in the Petri dish per trial for each of the experimental conditions. A video loop containing randomly distributed black circles of 0.3 mm radius was projected on a white background. This size was found to produce optimal responses to the stimulus as shown in Nagel et al. (2015). Swimming tadpoles were exposed to the moving circles for a period of 30 s per trial for a total of six trials per tadpole. The tadpole’s response to every circle encountered was analyzed blind to treatment, with frame-by-frame replay of recorded behaviors, by tracing each tadpole’s swimming path. Tadpoles were observed to freeze, swim in circles, and/or swim away by altering their direction, speed, or both when presented with stimuli. These responses were counted as visual reactions to the stimuli. Failure to alter the swimming path, move away from the circle or a lack of freezing behavior was considered a failure to respond. Experiments were performed during the light phase of the 12-h light-dark cycle. Treatments were identical to those of drug treatment and *in vivo* imaging studies with the exception that tadpoles were exposed to pharmacologic agents in rearing solution beginning at stage 45 and tested for the behavioral task 24 h later. The behavior of a total of 20–24 tadpoles was analyzed per condition from three independent experiments: Student’s *t*-tests and one-way ANOVA with Tukey’s multiple comparison tests were used for the statistical analysis of the data. Results of behavioral analysis were considered significant as follows: *p ≤ 0.05, ^**^p ≤ 0.005, ^***^p ≤ 0.001.

## Results

### CB_1_R expression in the retina and midbrain of *Xenopus* tadpoles

**FIGURE 1:**
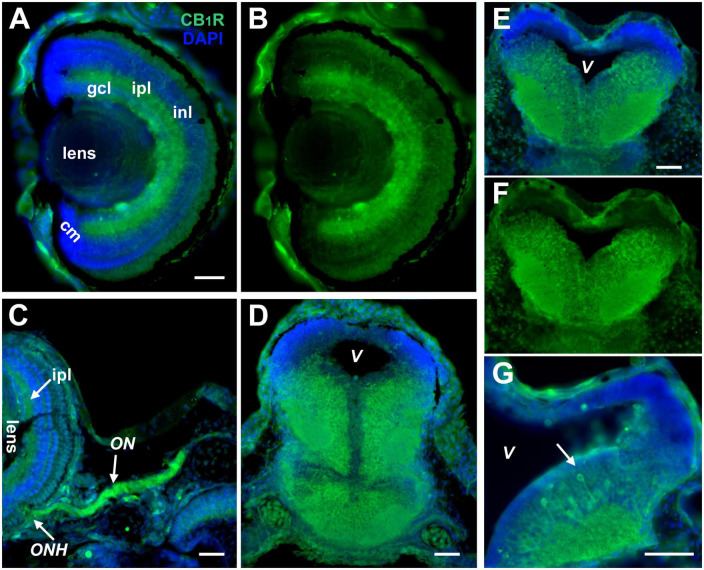
CB_1_R expression in the developing *Xenopus* visual system. **(A,B)** Coronal section of a retina of a stage 45 tadpole shows localization of strongest CB_1_R immunoreactivity (green) in the ganglion cell layer (gcl), inner nuclear layer (inl), and the inner plexiform layer (ipl). The cellular layers are clearly denoted by the DAPI staining in **(A)** (blue). Note the absence of CB_1_R immunoreactivity in the ciliary margin (cm). **(C)** Strong CB_1_R immunoreactivity is also observed in the optic nerve head (ONH) where RGC axons exit the retina, and along the optic nerve (ON). **(D–F)** CB_1_R expression in the brain is illustrated by the coronal sections of midbrain **(D)**, caudal midbrain **(E,F)** and rostral hindbrain **(G)** where CB_1_R immunoreactivity (green) localizes to cell bodies that lay medially and in the adjacent neuropil of stage 43 to stage 45 tadpoles. Note the individual neuronal cell bodies and processes projecting to the neuropil with strong CB_1_R immunoreactivity (**G**, arrow). In **(D,E,G)**, the DAPI staining highlights the lower CB_1_R immunoreactivity in the proliferative zones near the ventricle (V) and its absence in the dorsal-most portion of the brain. Scale bars = 50 μm. Similar patterns of CB_1_R immunoreactivity were obtained with two commercial antibodies to CB_1_R (**A,B**,**E–G**; Cayman Chemicals, and **C,D**; Calbiochem).

Studies have shown that the endocannabinoid receptor CB_1_R is dynamically regulated during the development of *Xenopus laevis* embryos, with its mRNA being detected as early as stage 28 and protein at stage 41 (Migliarini et al., 2006). To define cell-autonomous roles for the CB_1_ receptor during retinotectal development, we first set out to confirm CB_1_R expression in *Xenopus* at the time when retinotectal circuits begin to form. We performed immunohistochemistry on coronal sections of stage 42 and stage 45 tadpoles using two distinct polyclonal antibodies against CB_1_R that showed consistent immunoreactivity patterns. In the *Xenopus* eye, strong CB_1_R expression localized to the RGC, inner plexiform and inner nuclear layers (Figures 1A, B) in agreement with published studies (Miraucourt et al., 2016), as well as in the optic nerve (Figure 1C). CB_1_R immunoreactivity was also strongly localized to cell bodies and neuropil within the *Xenopus* midbrain and hindbrain (Figures 1D–F). As illustrated in Figure 1G, individual neuronal cell bodies and processes projecting to the neuropil could be distinguished by their strong CB_1_R immunoreactivity. Little CB_1_R immunoreactivity was detected near the ciliary margin of the retina, where retinal precursor cells localize at these stages (Figure 1A), and was similarly absent in the proliferative areas of the brain (Figures 1D–G). Thus, the expression patterns of CB_1_R in *Xenopus* are consistent with potential pre- and postsynaptic cell-autonomous roles in postmitotic neurons in the developing visual system.

### Cell-autonomous CB_1_R signaling impacts RGC axon morphology

**FIGURE 2:**
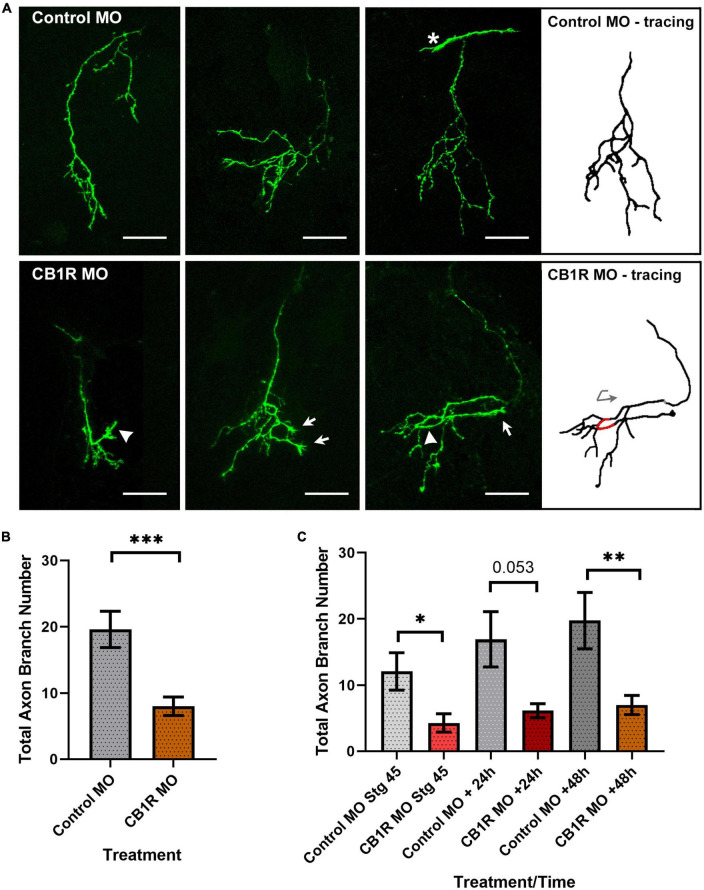
CB_1_R downregulation alters RGC axon morphology. **(A)** Projections of three individual RGC axons transfected with Control MO (top) or CB_1_R MO (bottom) imaged *in vivo* by two-photon confocal microscopy. While axons from RGCs transfected with the CB_1_R MO targeted normally within the tectal neuropil, their branching patterns and morphologies differed from those of RGCs in tadpoles transfected with Control MO. Qualitatively, axons from RGCs with CB_1_R knockdown had more branches that terminated in growth cones (arrows) and/or took abnormal turns (arrowheads). The tracing for the third CB_1_R MO sample axon better illustrates the abnormal turn (red; curved arrow) taken by the axon. The asterisk in the third Control MO sample points to the growth cone of a targeting RGC axon. Scale bars = 50 μm. **(B)** Quantitatively, axons from RGCs with CB_1_R knockdown had significantly fewer branches when compared to axons from RGCs transfected with Control MO 48 h after transfection (*n* = 23 axons per condition, one axon per tadpole). **(C)** When imaging over the course of 3 days, axon branch number was significantly lower in RGCs with CB_1_R MO knockdown at the initial imaging (stage 45), with a trend at the 24-h imaging interval (+24 h) and remaining significantly lower when compared to controls at the end of the imaging period (+48 h). Control MO *n* = 12; CB_1_R MO *n* = 11. Mean ± SEM. *p ≤ 0.05, **p ≤ 0.005, ***p ≤ 0.001.

A number of studies support roles for CB_1_R signaling during RGC differentiation and function in *Xenopus* as in other species (Martella et al., 2016; Miraucourt et al., 2016; Middleton et al., 2019; Elul et al., 2022). However, direct effects of CB_1_R -mediated endocannabinoid signaling in the development of visual central connectivity have not been demonstrated. Here, we examined potential CB_1_R cell-autonomous effects by downregulating its expression through targeted CB_1_R MO knockdown in tadpoles at the time when retinotectal connections form. Single-cell co-electroporation of lissamine-tagged Control or CB_1_R MO together with Alexa 488 dextran was used to downregulate CB_1_R expression at stage 43 and visualize individual RGC axons as they terminate in the optic tectum beginning at stage 45, 24 h after MO transfection. While axons from RGCs transfected with the CB_1_R MO targeted normally within the tectal neuropil, their branching patterns and morphologies differed from those of RGCs in tadpoles transfected with Control MO (Figure 2). Qualitatively, axons from RGCs with CB_1_R knockdown had more branches that terminated in growth cones and/or took abnormal turns (Figure 2A). When quantifying the total number of branches in axons imaged 48 h after transfection, axons from RGCs with CB_1_R knockdown had significantly fewer branches when compared to axons from RGCs transfected with Control MO (Control MO 19.61 ± 2.72 branches, CB_1_R MO 8.0 ± 1.41 branches; *n* = 23 axons per condition, with one axon imaged per tadpole; Figure 2B). To determine the time course of the knockdown effect, a smaller sample of tadpoles with targeted RGC MO knockdown were imaged over the course of 3 days, beginning at stage 45, 24 h after transfection. Axon branch number was significantly lower in RGCs with CB_1_R MO knockdown at the first imaging interval and remained low when compared to controls at the end of the observation period, while control RGC axons tended to increase their branch number over the course of 3 days (*n* = 12 axons for Control MO, *n* = 11 axons for CB_1_R MO, one axon imaged per tadpole; Figure 2C). These results demonstrate that presynaptic RGCs that express CB_1_Rs are capable to respond cell-autonomously to acute alterations in eCB signaling.

### Endocannabinoid levels and pharmacologic manipulation in *Xenopus* embryos and tadpoles

To further examine effects of altered endocannabinoid signaling on presynaptic retinotectal connectivity, we exposed tadpoles to pharmacologic agents starting at two distinct stages of development, at stage 38/39, when the first RGC axons travel to and begin to innervate the optic tectum (Holt and Harris, 1983), and at stage 45 when retinotectal connections actively form (Alsina et al., 2001). Tadpoles at these stages were evaluated for fatty acid content to determine endogenous levels and confirm the efficacy of the treatment. Tadpoles were treated with URB597 (2.5 μM), a selective inhibitor of FAAH, the enzyme that degrades AEA and related compounds, or JZL184 (2.5 μM), a potent and selective inhibitor of MAGL, the enzyme that blocks 2-AG hydrolysis. In control, vehicle-treated tadpoles, levels of AEA, palmitoylethanolamide (PEA) and oleoylethanolamide (OEA) were lower at stage 38 and increased by stage 45 (Table 1). URB597 treatment at stage 38 did not significantly change the levels of AEA, PEA, OEA or 2-AG (Table 1). However, at stage 45, URB597 treatment significantly increased AEA and OEA levels but did not change PEA or 2-AG levels when compared to vehicle or JZL184-treated tadpoles (Table 1), indicating the efficacy of the FAAH inhibitor treatment and suggesting that fatty acid synthesis and metabolism are dynamically regulated. Similar to AEA, levels of 2-AG were lower in stage 38 tadpoles than at stage 45 but were orders of magnitude higher than those of AEA at both stages (Stage 38; AEA approx. 20 pg/mg of protein, 2-AG approx. 470 ng/mg protein; Table 1). Interestingly, treatment of tadpoles with JZL184 significantly increased 2-AG levels at stage 38 when compared to vehicle or URB597-treated tadpoles but not at stage 45 (Table 1), again suggesting a differential, dynamic regulation 2-AG synthesis and metabolism during development. Levels of AEA, PEA or OEA were not significantly different in JZL184 treated tadpoles from those in vehicle-treated tadpoles at either of the two stages analyzed (Table 1), eliminating the possibility of off-target effects.

**TABLE 1:** Fatty acid analysis of tadpole samples.

	Fatty acid	Concentration	(pg/mg)	Concentration (ng/mg)
	AEA	PEA	OEA	2-AG
**Stage 38 treatment**				
DMSO	19.5 ± 7.5	986.0 ± 139	102.4 ± 13.1	466.2 ± 39.1
URB597	24.8 ± 4.8	1,231 ± 114	193.6 ± 33.9	325.3 ± 45.2
JZL184	48.16 ± 5.4	1,282 ± 243	216.9 ± 19.3	886.5 ± 133*
**Stage 45 treatment**				
DMSO	90.3 ± 23.2	2,012 ± 220	501 ± 13.6	1,263 ± 107
URB597	224.5 ± 53.5**	4,124 ± 1633	931 ± 225.2*	1,553 ± 363
JZL184	76.0 ± 21.5	3,074 ± 407	412.8 ± 83.5	940 ± 226

One-way and two-way ANOVA with Tukey’s multiple comparison tests show significant differences between JZL184 versus DMSO and URB597 treated tadpoles at stage 38 (*p ≤ 0.05), and URB597 versus DMSO and JZL184 treated tadpoles at stage 45 (**p ≤ 0.005). OEA levels are also significantly increased by the URB597 treatment at stage 45 (*p ≤ 0.05). No other significant difference among groups either at stage 38 or stage 45 was found. Mean ± SEM.

### Rate of axon branching is selectively increased after URB597 treatment in actively branching RGCs

**FIGURE 3:**
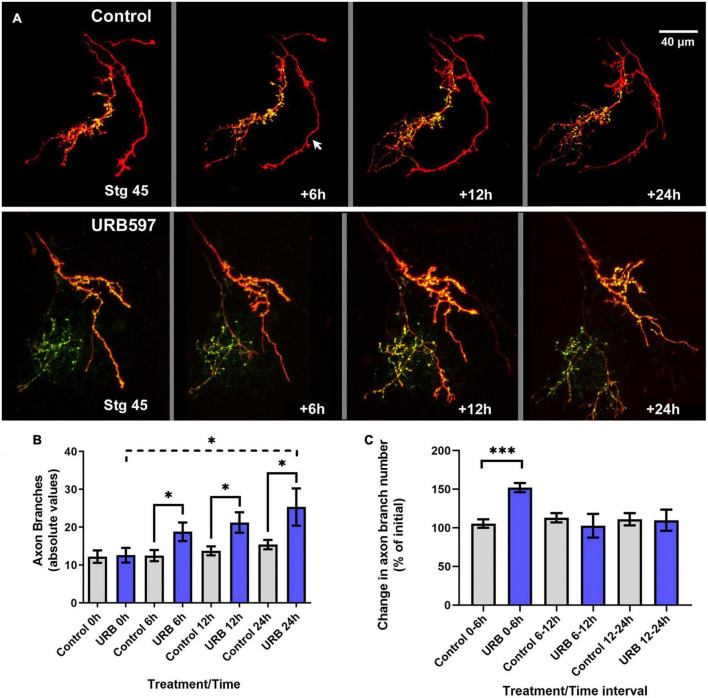
Rapid and transient branching response by RGC axons to acute tectal URB597 treatment. **(A)** Tadpoles with RGC axons co-expressing tdTomato and GFP-synaptobrevin received an acute, localized injection of URB597 into the optic tectum at stage 45 and were imaged by confocal microscopy 6, 12 and 24 h after initial imaging (stg 45, time 0 h). Two sample arbors in a control tadpole (top panel) and in a tadpole with tectal injection of URB597 (bottom panel) illustrate the changes in dynamic branching and in the number and localization of GFP-labeled pre-synaptic sites in the axon arbors. In the control sample, one axon expresses tdTomato only (arrow). Scale bar = 40 μm. **(B)** Quantitative analysis of total branch number shows that RGC axons had significantly more branches in the URB597-treated tadpoles during first 6 h after treatment than RGC axons in control tadpoles, a significant difference that was maintained for 24 h. **(C)** The rate of axon branching, expressed as the change in branch number per imaging interval, was significantly higher during the first 6 h after URB597 tectal injection and returned to a similar rate to those in control tadpoles at the 6–12- and 12–24-h imaging intervals. Control *n* = 12 axons, URB597 *n* = 7 axons. Analysis by Student’s *t*-tests. Mean ± SEM. *p ≤ 0.05, ***p ≤ 0.001.

**FIGURE 4:**
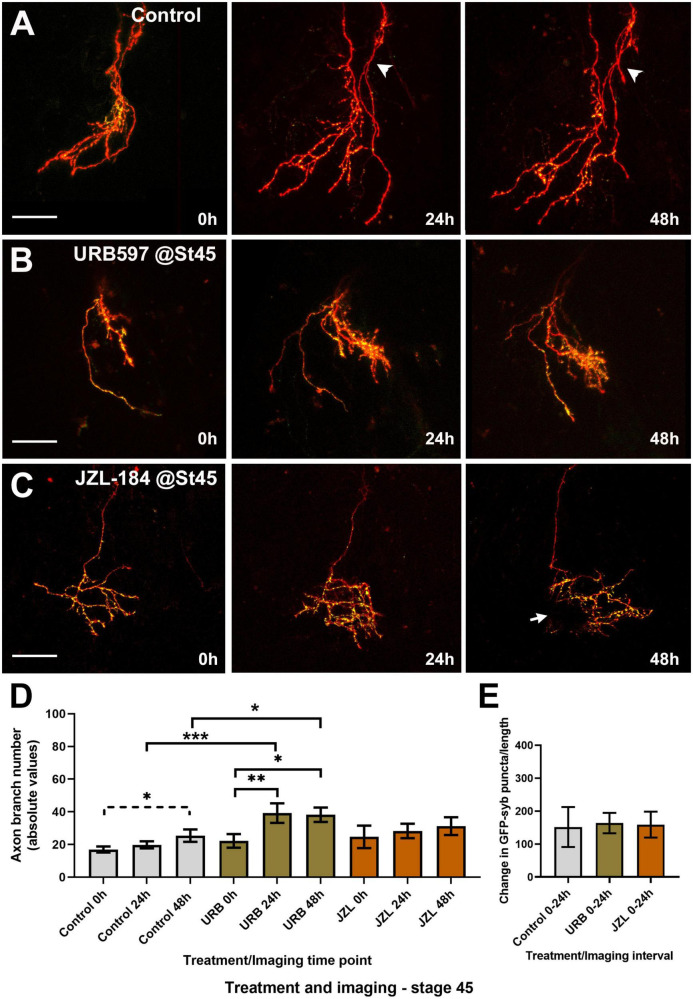
Global URB597 treatment at stage 45 increases the complexity of actively branching RGCs *in vivo*. **(A–C)** Sample RGC axons in stage 45 tadpoles transfected with tdTomato and GFP-synaptobrevin plasmids. Tadpoles imaged *in vivo* at stage 45 before (0 h), and 24, and 48 h after vehicle (**A**; Control), URB597 **(B)**, or JZL184 **(C)** bath treatment. RGC axons gradually increased their number of branches over a period of 48 h. Arrowhead in **(A)**, points to a second axon. Arrow in **(C)** points to an area of the arbor obscured by a pigment cell. Scale bars = 50 μm. **(D)** Quantitative analysis of branch number in RGC axons in URB597-treated tadpoles shows a faster and significant increase in branch number during the first 24 h of treatment, resulting in a higher number of branches by 24 and 48 h vs. controls (*n* = 10 axons per condition). No significant difference in the number of branches was observed for axons in JZL184 treated tadpoles at any observation interval when compared to axons in control tadpoles. **(E)** The density of GFP-synaptobrevin puncta per arbor was calculated as the number of puncta per 20 μm. No significant difference in puncta density before and 24 h after treatment (change in GFP-synaptobrevin puncta/length 0–24 h) was observed in axons from URB597- or JZL184-treated tadpoles when compared to controls. Analysis by ANOVA with Sidak’s multiple comparisons tests and Student’s *t*-test. Mean ± SEM, *p ≤ 0.05, **p ≤ 0.005, ***p ≤ 0.001.

To further evaluate how endocannabinoid receptor activation impacts RGC axon morphology at the time that retinotectal connections form, we examined potential changes in RGC axon targeting, branching and synaptic differentiation upon changes in eCB levels by imaging individual RGC axons co-expressing tdTomato and GFP-synaptobrevin as they innervate the tectal neuropil in stage 45 tadpoles. We used URB597 to increase endogenous AEA levels in the brain. Microinjection of a single, acute dose of URB597 (1 nL of 50 μM solution) into the optic tectum resulted in a rapid change in RGC axon morphology at the target within the first 6 h after treatment when compared to vehicle-injected controls (Figures 3A, B). The number of branches and the rate of RGC axon branching (change in branch number) in URB597 treated tadpoles was significantly higher than in controls during the first 6 h after treatment (0–6 h; Control 105.5 ± 5.4%; URB597 152.1 ± 5.9%, *p* = 0.0002; Figure 3C). While URB597-treated axons continued to branch and increased their complexity at the 12- and 24-h imaging time points (Figure 3B), the rate of axon branching returned to that of control levels by the 12-h imaging time point (Figure 3C). Thus, the effects of acute increase in eCB signaling, as a result from blocking hydrolysis of AEA and related eCBs by the FAAH inhibitor, were rapid and involved a significant remodeling of axon arbors. To further evaluate effects of a more chronic treatment, we exposed tadpoles to URB597 for a period of 48 h at stage 45 when axons actively branch. Immediately after initial imaging, we reared stage 45 tadpoles with RGCs expressing tdTomato and GFP-synaptobrevin in URB597 (2.5 μM in rearing solution) for 2 days. RGC axons in URB597-treated tadpoles significantly increased their number of branches during the first 24 h of treatment when compared to controls, resulting in RGC axons with a significantly higher branch number than those in control tadpoles by 24 and 48 h (Figures 4A, B, D). The observation that RGC axons in URB597-treated tadpoles showed a fast response to URB597 treatment (at 24 h) but did not further increase their number of branches by 48 h, again indicate a rapid effect of the drug treatment. Imaging of RGC axons also showed that during the first 24 h of treatment, GFP-synaptobrevin puncta were added at rate to maintain the normal density of presynaptic sites in the more elaborate RGC axon arbors (Figures 4A, B, E). Specifically, as axons in URB597-treated tadpoles branched more rapidly during the first 24 h of treatment (Change in branch number 0–24 h: Control 146.7 ± 16.4%, URB597 242.3 ± 60.2%; *p* = 0.043, not shown graphically), the number of GFP-synaptobrevin puncta in those axons increased at a rate to maintain a similar density in the more branched arbors when compared with axons from control tadpoles (Change in GFP-syb puncta/length 0–24 h: Control 151.8 ± 60.6%; URB597 163.7 ± 31.1%; *p* = 0.854; Figure 4E).

**FIGURE 5:**
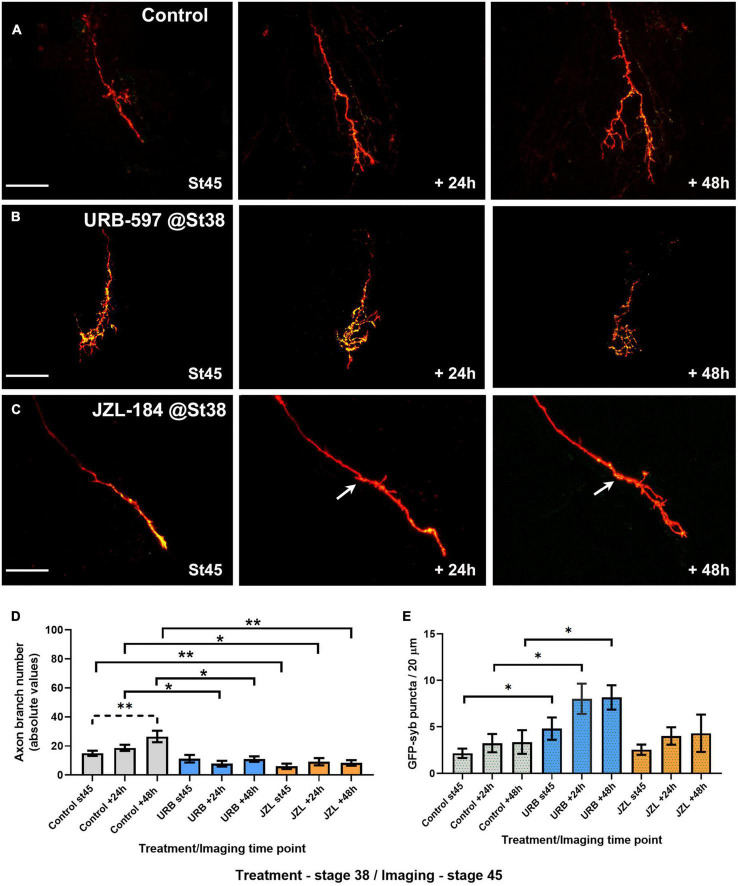
URB597 or JZL184 treatment during early axon pathfinding and targeting results in RGC axons with much simpler morphologies as they branch at their target. Tadpoles were treated with URB597 or JZL184 beginning at stage 38 until stage 45, when tadpoles with RGC axons expressing tdTomato and GFP-synaptobrevin were imaged for three consecutive days in the absence of the drug. **(A–C)** Sample RGC axons in tadpoles treated with vehicle solution (**A**; Control), URB597 **(B)** or JZL184 **(C)** imaged *in vivo* by confocal microscopy at stage 45, and 24, and 48 h after first imaging. Arrow in **(C)** points to the first branching point of the axon. Scale bars = 50 μm. **(D)** Quantitative analysis of branch number in RGC axons of tadpoles exposed to the drugs from stage 38 to stage 45 showed that RGC axons in both JZL184 and URB597-treated tadpoles failed to increase their branch number over the course of 48 h when compared to controls (*n* = 11 axons per condition). Axons in control-treated tadpoles significantly increased their branch number over the 48-h imaging period. **(E)** The density of GFP-synaptobrevin puncta per arbor was calculated as the number of puncta per 20 μm. When compared to controls, axons in URB597-treated tadpoles had a significant higher puncta density at all imaging intervals. Analysis by ANOVA with Sidak’s multiple comparisons tests. Mean ± SEM, *p ≤ 0.05, **p ≤ 0.005.

To evaluate whether altered endocannabinoid levels and signaling influence RGC axons not only as they branch but also as they travel to the tectum and before they begin to branch, tadpoles co-electroporated with tdTomato plus GFP-synaptobrevin expression plasmids were then treated with URB597 beginning at stage 38 and every 24 h after for 2 days until they reached stage 45. Tadpoles were then transferred to fresh rearing medium without the drug and imaged at that stage (stage 45; 0 h) and 24 and 48 h later. Treatment of young tadpoles with URB597 resulted in RGC axons with similar morphologies to those in aged matched control tadpoles by stage 45 but failed to increase their branch number over the course of 2 days (Figures 5A, B). In contrast to the effects of treatment beginning at stage 45, the number of axon branches was significantly lower in RGCs at the 48-h imaging time point in tadpoles treated with URB597 at stage 38 when compared to RGCs in control tadpoles at the same stage (Figure 5D). Surprisingly, even though chronic treatment with URB597 in tadpoles at stage 38 resulted in RGC axons with more immature axon morphologies at stage 45, those RGC axons had a significantly higher density of GFP-synaptobrevin labeled presynaptic sites than axons in control tadpoles at all imaging intervals (GFP-syb puncta/20 μm: Control 0 h 2.18 ± 0.5, URB597 0 h 4.8 ± 1.1; Control 24 h 3.26 ± 0.9, URB597 24 h 8.02 ± 1.6; Control 48 h 3.36 ± 1.2, URB597 48 h 8.17 ± 1.3, *p* ≤ 0.05, *n* = 9 axons for control, *n* = 7 axons for URB597, Figure 5E). Together these results indicate that endogenous AEA participates in axon branching and synaptic differentiation and that differential effects at distinct stages of axon development may reflect differential activation and/or desensitization of CB_1_R or of the eCB system during development.

### Early JZL184 treatment interferes with axon branching and induces pathfinding errors in RGC axons as they target in the optic tectum

**FIGURE 6:**
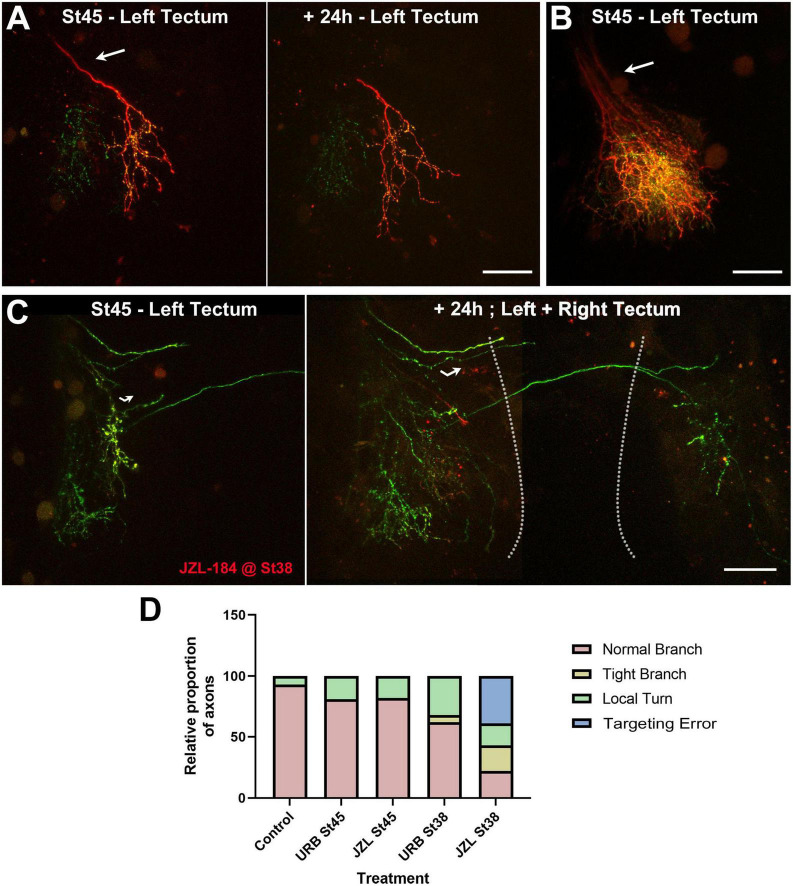
Targeting errors in arborizing RGC axons induced by early JZL184 treatment. **(A–C)** Confocal projections of RGC axons branching in the optic tectum of control **(A,B)** and JZL184-treated **(C)** tadpoles imaged at stage 45. **(A,B)** The examples of control tadpoles with individual axons at stage 45 and +24 h **(A)**, or multiple axons at stage 45 **(B)** illustrate how RGC axons enter the optic tectum through the optic tract (arrows) with similar directionality before they branch. **(C)** Confocal images of the optic tectum of a tadpole treated with JZL184 beginning at stage 38 show RGC axons with apparent targeting errors within the contralateral hemisphere at stage 45 (left panel, curved arrow), and reveal abnormal crossing of axons to the ipsilateral hemisphere 24 h after initial imaging (right panel; stitched image). The dashed lines delineate the two midbrain hemispheres. Scale bars = 100 μm. **(D)** While quantitatively RGC axon arbors in tadpoles treated with URB597 and JZL184 at stage 38 differ in the overall number of branches they possessed at stage 45 (Figure 5), in proportion axon arbors were also observed to make abnormal turns (local turns) within the neuropil and/or to branch more locally (tight branch) when treatment began at stage 38. Moreover, in proportion more RGC axons in tadpoles treated with JZL184 projected aberrantly, showing abnormal ipsilateral crossing (targeting errors). Control *n* = 14, URB597 at stage 45 *n* = 16, JZL184 at stage 45 *n* = 11, URB597 at stage 38 *n* = 19, JZL184 at stage 38 *n* = 28. Statistical analysis by chi-square, difference in outcome among groups *p* ≤ 0.0001.

To evaluate potential differential effects of AEA and 2-AG-mediated endocannabinoid signaling on RGC axons, a similar protocol was used to increase endogenous 2-AG levels by adding JZL184, a MAGL inhibitor, to the rearing solution either at stage 45 or stage 38. In contrast to URB597 treatment, treatment of stage 45 tadpoles with JZL184 over the course of 2 days resulted in RGC axons with similar number of branches at all observation time points when compared to controls (Figures 4C–E). While no significant changes in RGC axon branch number or branch dynamics were observed in tadpoles exposed to JZL184 at stage 45, axons were simpler and less branched than controls when treatment was initiated at stage 38. In JZL184-treated tadpoles, RGC axon branch number was significantly lower than controls at the 48-h imaging time point (Figures 5C, D). Moreover, we observed that treatment of tadpoles with JZL184 at stage 38 resulted in RGC axons with more branching and axon pathfinding errors, including abnormal crossing to the ipsilateral optic tectum (Figures 6A–C). Quantitatively RGC axon arbors in stage 38 JZL184-treated tadpoles did not differ in the overall number branches they possessed from those in URB597 treated tadpoles, however, axon branches made more abnormal turns within the neuropil and/or branched more locally in JZL184 treated tadpoles than in URB597-treated and control tadpoles (Figure 6D). The density of GFP-synaptobrevin labeled presynaptic sites in axons from JZL184-treated tadpoles was not significantly different from control tadpoles at any of the imaging intervals (GFP-syb puncta/20 μm: Control 0 h 2.18 ± 0.5, JZL184 0 h 2.56 ± 0.55; Control 24 h 3.26 ± 0.9, JZL184 24 h 4.02 ± 0.9; Control 48 h 3.36 ± 1.2, JZL184 48 h 4.31 ± 1.8, *p* ≤ 0.05, *n* = 9 axons per condition, Figure 5E). Together, these results indicate that differential AEA- and 2-AG-mediated endocannabinoid signaling at distinct times of development impacts RGC axon arbor morphological and synaptic differentiation at the time that active *Xenopus* retinotectal synaptic connections are made.

### Cell-autonomous CB_1_R signaling also contributes to the structural differentiation of postsynaptic tectal neurons

**FIGURE 7:**
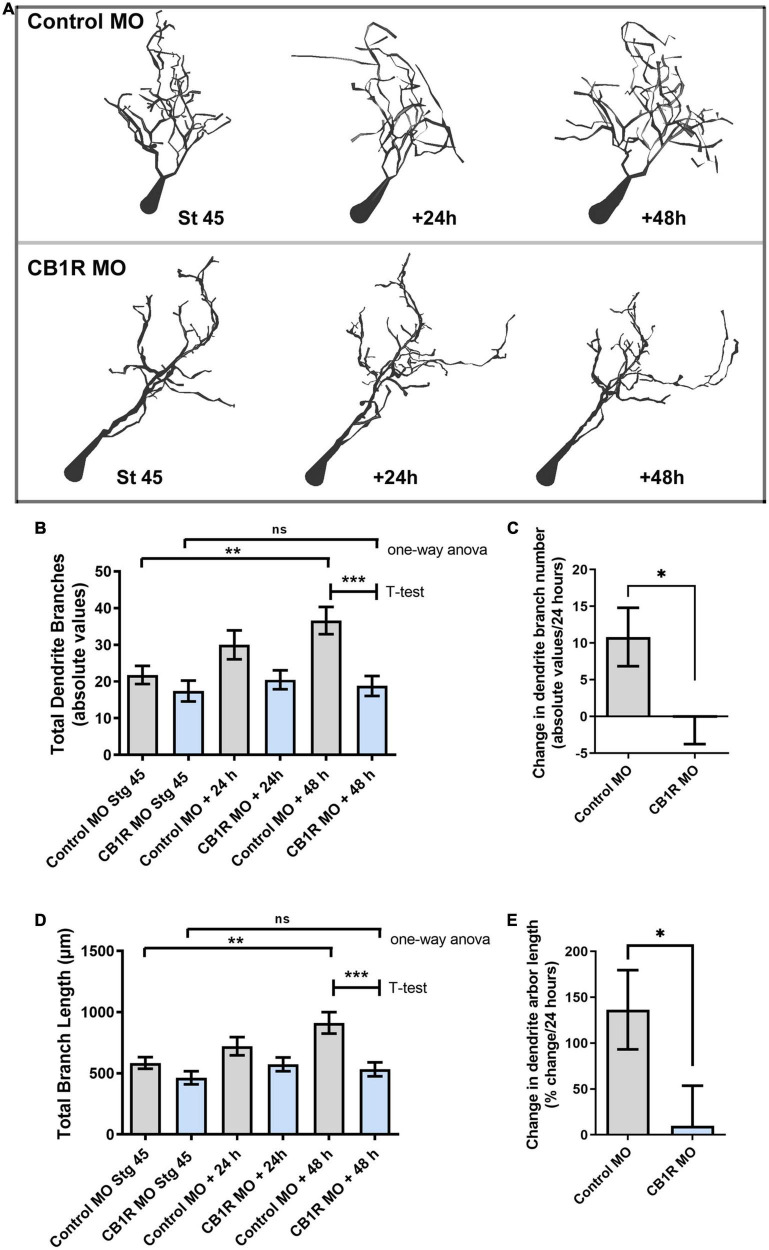
Single-cell CB_1_R knockdown decreases the branching and growth of tectal neurons *in vivo*. **(A)** Tracings of sample neurons in stage 45 tadpoles transfected with Alexa 488 dextran and lissamine-tagged Control MO or CB_1_R MO and imaged *in vivo* by two-photon confocal microscopy over the course of 3 days. Dendritic arbors were digitally reconstructed in three-dimensions using the Neuromantic tracing software and rendered for illustration purposes using Adobe Photoshop. **(B)** Dendritic arbors of neurons with CB_1_R MO had a similar number of branches as Control MO neurons at stage 45 after but possessed a significantly a lower number of branches than controls by the 48-h imaging time point. **(C)** Quantifying the rate of branch addition as the change in branch number in every 24 h-imaging intervals showed that neurons transfected with Control MO increased their complexity by adding new branches, neurons transfected CB_1_R MO failed to increase the complexity of their dendritic arbor. **(D,E)** Quantification of total dendritic arbor length reveals that tectal neurons with CB_1_R MO fail to increase their dendritic arbor length **(D)** and grow at a lower rate **(E)** than Control MO transfected neurons (*n* = 29 neurons per condition). Statistical analysis by one-way ANOVA and Student’s *t*-test. Mean ± SEM. *p ≤ 0.05, **p ≤ 0.005, ***p ≤ 0.001.

Studies have shown that CB_1_R expression is high in glutamatergic projection neurons during development and then decreases as glutamatergic circuits mature (Vitalis et al., 2008), consistent with our observation of a CB_1_R -mediated presynaptic action on RGCs in a differentiating retinotectal circuit. To also examine whether CB_1_R signaling can directly impact postsynaptic neurons in the visual system, we examined the morphological differentiation of tectal neurons with targeted CB_1_R knockdown (Figure 7). Single-cell co-electroporation of lissamine-tagged Control or CB_1_R MO together with Alexa 488 dextran in the brain of stage 43 tadpoles was used to downregulate CB_1_R expression and visualize individual tectal neurons beginning at stage 45, 24 h after MO transfection. At stage 45, tectal neurons with CB_1_R knockdown had a similar dendrite branch number as control MO-transfected neurons (Control MO 21.74 ± 2.47 branches, *n* = 45 neurons, CB_1_R MO 17.40 ± 2.85 branches, *n* = 42 neurons; Figure 7B). However, while control tectal neurons continued to branch and significantly increase their number of branches 24 and 48 h after the first imaging, neurons with CB_1_R MO knockdown failed to increase their branch numbers and length over the course of 2 days (Figures 7C, E), and were significantly simpler than controls by 48 h (Figures 7B, D). Thus, altering CB_1_R-mediated endocannabinoid signaling can also impact dendritic morphology and connectivity of postsynaptic tectal neurons cell-autonomously, although with a different time scale (significant at the 48-h imaging time point, Figures 7B, D) when compared to the cell-autonomous effects on presynaptic RGCs (significant at the first imaging time point, Figure 2C).

### Altering AEA levels enhanced visually guided responses

**FIGURE 8:**
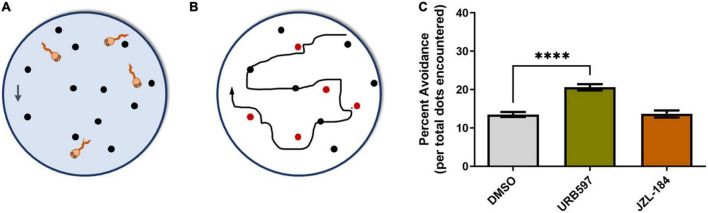
URB597 treatment enhances visually guided responses. **(A)** Schematic of the visual avoidance task. Tadpoles at stage 45, treated with vehicle (0.1% DMSO in rearing solution), URB597 or JZL184, were tested for their ability to alter their swimming behavior upon encountering moving dots (arrow depicts downward movement of dots). **(B)** The tadpole’s swimming path and responses to advancing stimuli (black and red circles) were tracked and analyzed. A tadpole freezing response upon coinciding with the stimulus (red circles) or changing its swimming direction and speed was considered active avoidance. **(C)** Tadpoles treated with URB597 had increased avoidance responses to the presentation of the stimulus 24 h post-treatment when compared to vehicle treated controls. Control *n* = 24 tadpoles, URB597 *n* = 20 tadpoles, JZL184 *n* = 20 tadpoles. Mean ± SEM. ****p ≤ 0.0001.

To determine the functional consequence of altered cannabinoid signaling during active RGC axon branching and synapse formation, and to correlate structural changes with functional changes at the same developmental stage, we used a visually-guided behavioral assay to measure responses to visual stimulation. Tadpoles at stage 45 were treated with vehicle (0.1% DMSO in rearing solution), URB597 (2.5 μM) or JZL184 (2.5 μM) for 24 h prior to behavioral testing. The tadpoles’ response to advancing stimuli (video loop of moving black dots) was measured and quantified as percent positive responses per total interactions with potential stimuli (see section “Materials and methods”). The avoidance response to stimulus in tadpoles treated with URB597, which would increase AEA levels, was significantly higher than that of tadpoles exposed to vehicle control or JZL184 for 24 h (Figure 8). Thus, structural changes in RGC axon arbors as caused by the URB597 treatment (Figures 3, 4) correlate with functional behavioral changes in visual responses to stimulus.

## Discussion

Our studies characterized the differential effects of eCBs on differentiating RGC axons at distinct times of development *in vivo*, demonstrating not only cell-autonomous effects of CB_1_R signaling on RGCs, but also that global and rapid changes in AEA and 2-AG levels and signaling in developing tadpoles can differentially influence RGC axon growth and connectivity in the intact developing organism.

Developmental studies have shown that CB_1_R is high evolutionary conserved among species and is highly expressed in the developing brain of multiple vertebrate species, from zebrafish to mammals (Lam et al., 2006; Martella et al., 2016; da Silva Sampaio et al., 2018). *Xenopus laevis* CB_1_R has 74% nucleotide sequence identity and 83% amino acid sequence identity with human CB_1_R (Cottone et al., 2003). In *Xenopus*, *cnr1* expression is relatively low from gastrula to neurula stages and increases five-fold by stage 34–35, during the early tailbud stage (Zheng et al., 2015). CB_1_R knockdown in early *Xenopus* embryos, at the four-cell stage, induces developmental eye defects by stage 41, supporting a role for CB_1_R signaling in retinal development and differentiation (Zheng et al., 2015). Moreover, recent studies have shown that exposure of whole brain preparations of young tadpoles to CB_1_R agonists alter the growth cone structure of RGCs as they travel through the optic tract (Elul et al., 2022), while pharmacological activation of the CB_1_R in *Xenopus* tadpoles at later stages increases RGC firing response to visual stimulation (Miraucourt et al., 2016). Functional analyses in zebrafish also support a role for eCBs in normal vision (Martella et al., 2016). Using *Xenopus*, we provide further evidence that endocannabinoids can differentially impact the structure and connectivity of RGCs at distinct times of development and that RGCs are capable to respond to changes in cannabinoid signaling cell-autonomously.

While CB_1_R has been detected in embryonic and postnatal neuronal progenitors in rodents, expression levels in differentiating and migrating progenitors in all species is very low compared to expression levels in newly differentiated neurons (Harkany et al., 2008a; Vitalis et al., 2008; Gaffuri et al., 2012). In agreement with observation of CB_1_R enrichment in differentiating neurons, our immunohistochemical studies showed that the eCB receptor CB_1_R is expressed in *Xenopus* tadpoles at higher levels in differentiated postmitotic neurons in the visual system (Figure 1 and see Miraucourt et al., 2016). In stage 45 tadpoles, a time when functional visual circuit connections form, CB_1_R immunoreactivity is absent in precursor cells near the ventricle and in the dorso-caudal midbrain but is highly expressed in newly differentiated cells in the midbrain and in the tectal neuropil. In retina, CB_1_R immunoreactivity is observed from time when RGCs extend axons to the brain (stage 42) to when they make functional synaptic connections with tectal cells (stage 45 to 47). Thus, the expression patterns of CB_1_R in the *Xenopus* visual system indicate that postmitotic RGCs can directly respond to CB_1_R signaling similar to those in mammals and other vertebrate species.

Similar to effects on proliferation, the effects of CB_1_R activation and eCB signaling on neurite outgrowth have mostly been analyzed using neuroblastoma-derived cell lines and primary neuronal cultures, with somewhat conflicting results (Gaffuri et al., 2012). For example, some studies have shown that CB_1_R activation has a positive effect on FGF2 and N-cadherin mediated neurite outgrowth of neurons in culture, an effect that may be mediated by 2-AG activity (Williams et al., 2003). In contrast, other groups have shown negative effects of CB_1_R activation on BDNF-induced neurite outgrowth in inhibitory neurons in culture as well as in axon and dendrite growth in CB_1_R-transfected neurons *in vitro* (Berghuis et al., 2005; Vitalis et al., 2008). Evidence from pyramidal neurons in culture treated with eCB agonists (AEA, ACEA) and inverse agonists (AM251) indicates that neurons require an eCB tone to initiate axonal polarization and that eCB signaling interferes with axon branching (Mulder et al., 2008). Other studies have shown that THC can affect cytoskeletal dynamics by modulating the expression of microtubule binding proteins that are necessary for axon growth (Tortoriello et al., 2014), while evidence also indicates that acute CB_1_R activation results in rapid contraction of the neuronal actomyosin cytoskeleton in hippocampal cells in culture (Roland et al., 2014). Pharmacologic studies also show that CB_1_R agonists attenuated activity-dependent remodeling of dendritic spines in mature cortical neurons in culture (Njoo et al., 2015). Thus, while most studies implicate the eCB system in establishing and maintaining a polarized morphology in developing neurons and in axon growth through cytoskeletal modifications, it remains unclear how developing neural circuits are dynamically shaped in the intact developing embryo, again highlighting the need to examine effects in more natural physiological settings.

Previous studies from our laboratory on the analysis of RGC axon growth and targeting demonstrate differential *in vivo* versus *in vitro* responses of RGC axon growth cones to axon guidance molecules. Specifically, by imaging RGC axon growth cones in Stage 40 *Xenopus* tadpoles right before they begin to arborize, we showed that netrin-1 impacts early axon arbor differentiation by altering branching responses at their optic tectal target and inhibiting growth cone advancement within the target (Shirkey et al., 2012). These effects on RGC axons were not recapitulated in culture on growth cones exposed to netrin-1 (Shirkey et al., 2012). Thus, the differential *in vivo* versus *in vitro* effects indicates that neurons need to integrate multiple cues in their local *in vivo* environment to modulate axon elongation, targeting and branching. Here, we demonstrate that both single-cell downregulation of CB_1_R expression as well as acute and chronic alterations in eCB levels in intact *Xenopus* tadpoles during RGC axon targeting and branching significantly impact RGC axons. Cell-autonomous downregulation of CB_1_R in postmitotic RGCs at the time they terminate in the optic tectum interfered with normal axon branching and resulted in RGC axons that abnormally retained terminal growth cones. Thus, negative regulation of CB_1_R function resulted in similar but less penetrant phenotypes to those observed on RGC axons after cell-autonomous alterations in BDNF-mediated TrkB signaling (Marshak et al., 2007), a molecular pathway that has been linked to eCB signaling (Maison et al., 2009; Yeh et al., 2017), but more penetrant phenotypes than after downregulation of DCC-mediated netrin signaling (Manitt et al., 2009; Shirkey et al., 2012), a mechanism that has been linked to CB_1_R -mediated growth cone steering (Argaw et al., 2011).

A role for eCB signaling in axon fasciculation and pathfinding has been suggested based on observations of diffuse thalamocortical and corticothalamic tracts in adult CB_1_R knockout mice and fasciculation deficits in callosal and corticofugal projections in neonatal CB_1_R knockout mice using fiber bundle analyses (Mulder et al., 2008; Wu et al., 2010). These analyses of mutant mice, while demonstrating that eCB signaling is important for axon growth, were not able to unequivocally demonstrate whether effects on axon fasciculation are independent of neural differentiation and did not distinguish from other potential global or time-dependent effects on growing axons. In the current studies, brain microinjection of URB597 resulted in a rapid increase in RGC axon branch number at the target within the first 6 h after treatment when compared to vehicle-injected controls (Figure 2). The effect on axon branch number was rapid, with axons then continuing to remodel at a normal rate and increasing their number of branches by 12 and 24 h. Thus, the effects of acute increase in eCB signaling, as would result from blocking hydrolysis of AEA and related eCBs by the FAAH inhibitor, are rapid and seem to involve a significant remodeling of axon arbors. These rapid effects are supported by the effects of prolonged, global treatment in tadpoles exposed to URB597 for 48 h. URB597 treatment at stage 45 resulted in a significant increase in the rate of axon branching 24 h after initial treatment, similar to the effect we observed after acute treatment but with a longer onset. In contrast, axon branch number was significantly lower over time in tadpoles that were treated with URB597 beginning at stage 38 and imaged at stage 45 when compared to controls as axons failed to branch. However, even though axons remained simpler than controls when tadpoles were treated with URB597 early as they path-find, the density of GFP-synaptobrevin labeled presynaptic sites was significantly higher than that in control axons. Thus, while the early increase in AEA levels and signaling interfered with subsequent axon branching at the target, it also positively impacted synapse number.

Neuronal responses to altered eCB levels and signaling may depend on the expression levels of key enzymes responsible for their synthesis and degradation and on the local environment where the neuron grows (Aguirre et al., 2019). FAAH is expressed in the retina of multiple vertebrate species (Yazulla et al., 1999; Glaser et al., 2005), and the enzymes responsible for the synthesis (DAGLα) and the degradation (MAGL) of 2-AG are expressed in multiple rodent retinal cell types during development, including RGCs (Cecyre et al., 2014). Developmental expression of both FAAH and MAGL has been characterized in zebrafish larva at stages when retinotectal connections are made, paralleling CB_1_R expression and function in vision in this same species (Martella et al., 2016). Even though tissue-specific expression of AEA or 2-AG biosynthetic and catabolic enzymes has not been directly demonstrated in *Xenopus laevis*, FAAH and DAGLα transcripts can be detected in developing embryos and tadpoles (Session et al., 2016). In *Xenopus*, AEA activation of the CB_1_R has been demonstrated to occur as early as stage 41 (Migliarini et al., 2006), while bath application of CB_1_R agonists has been found to directly enhance RGC excitability within the retina of stage 45 tadpoles without the need for retinotectal feedback (Miraucourt et al., 2016). Here, we used similar protocols to increase endogenous AEA levels with URB597 or 2-AG levels by adding JZL184, a MAGL inhibitor, in tadpoles at stage 38 or stage 45. While no significant changes in axon branch number or branch dynamics were observed in tadpoles exposed to JZL184 at stage 45, axons were simpler and less branched than controls when treatment was initiated at stage 38. Moreover, early JZL184 treatment induced significant pathfinding errors in a subset of RGC axons at the target, abnormally crossing to the opposite side of the optic tectum. These effects coincide with the timing at which JZL184 treatment significantly increases endogenous 2-AG levels in tadpoles, thus supporting the idea that presynaptic RGC axons that express CB_1_Rs are capable to respond to acute alterations in eCB signaling but that effects of AEA and 2-AG are distinct and depend on the developmental stage. That AEA and 2-AG differentially impact *Xenopus* RGC axon navigation and branching through direct CB_1_R signaling on RGCs, however, remains to be established.

It is likely that pharmacologic and genetic manipulations which alter eCB levels and signaling impact not only RGCs but most neurons and glia throughout the CNS. The effects of pharmacologic manipulation in young tadpoles may also depend on the global ability of the drug treatment to affect multiple/different circuits at distinct times of development, as suggested by the differential effects of the drug treatments on the levels of AEA, 2-AG and other fatty acid metabolites on the whole tadpole organism. However, our studies show that downregulation of CB_1_R in single, developing pre- and postsynaptic neurons in the otherwise intact brain of living animals is sufficient to alter their connectivity at the time that synaptic connections are made. That altered connectivity is induced by time-dependent changes in eCB levels and signaling is also supported by our observations that a short exposure to drugs that interfere with FAAH function, and thus increase eCB levels, results in functional changes in the visual circuit as measured by our visual avoidance task; results that are similar to those of more chronic manipulations in *Xenopus* tadpoles (Miraucourt et al., 2016). Moreover, effects of JZL184 treatment on misrouting of targeting *Xenopus* RGC axons support a role for eCB signaling in axon navigation effects, as shown for CB_1_R knockout mice where axon fasciculation of corticothalamic and thalamocortical axons is altered possibly through altered 2-AG signaling (Wu et al., 2010). Similarly, pathfinding errors that include increased contralateral crossing of spinal axons in zebrafish larvae with CB_1_R MO knockdown (Watson et al., 2008), support a role for eCB-mediated CB_1_R signaling in guiding developing axons. Understanding how cannabinoid signaling can induced cell-autonomous changes in connectivity in the developing brain is of significance, especially as negative regulation of cannabinoid receptor expression and function has been correlated with THC/cannabis consumption (Tortoriello et al., 2014; Silva et al., 2015). Our single-cell analysis of CB_1_R function in *Xenopus* expands our understanding of how cell-autonomous CB_1_R-mediated eCB signaling in single neurons, and how global alterations in eCB levels in a developing embryo, can impact pre- and postsynaptic neuronal connectivity in the intact brain.

## Data availability statement

The raw data supporting the conclusions of this article will be made available by the authors, without undue reservation.

## Ethics statement

This animal study was reviewed and approved by the Institutional Animal Care and Use Committee of the University of California, Irvine (Animal Welfare Assurance Number: A3416–01).

## Author contributions

SC-C, RDR, and RAS conceived the project. RDR and RGS conducted knockdown and imaging experiments. RDR, EG, and JM conducted pharmacologic experiments. ME conducted behavioral experiments. RAS conducted imaging and immunohistochemical experiments. RDR, EG, JM, ME, KD, and SC-C analyzed data and the Impact of Cannabinoids Across Lifespan (ICAL) Center for the Study of Cannabis at UCI ran the LC/MS analysis. SC-C wrote the manuscript with input from all authors. All authors contributed to the article and approved the submitted version.
